# CARDIOVASCULAR-DERIVED CELL-FREE DNA: ASSOCIATIONS WITH FRAILTY AND AGING-RELATED COMORBIDITIES

**DOI:** 10.1093/geroni/igae098.1722

**Published:** 2024-12-31

**Authors:** Lolita Nidadavolu, David Sosnowski, Nikita Sivakumar, David Bennett, Jude Phillip, Brion Maher, Esther Oh, Peter Abadir

**Affiliations:** Johns Hopkins University, Baltimore, Maryland, United States; Johns Hopkins University, Baltimore, Maryland, United States; Johns Hopkins University, Baltimore, Maryland, United States; Rush University Medical Center, Chicago, Illinois, United States; Johns Hopkins University, Baltimore, Maryland, United States; Johns Hopkins University, Baltimore, Maryland, United States; Johns Hopkins University, Baltimore, Maryland, United States; Johns Hopkins University, Baltimore, Maryland, United States

## Abstract

Cell-free genomic DNA (cf-gDNA) fragments are released into the bloodstream following cell death processes and higher levels are associated with age-related physical and cognitive decline. It remains unclear which tissues release cf-gDNA fragments in community-dwelling older adults with frailty. Serum cf-gDNA from 190 participants in the Religious Orders Study and Rush Memory and Aging Project (ROS-MAP) had DNA methylation performed via Illumina Methylation EPIC array. Hierarchical clustering derived distinct sub-populations based on cf-gDNA tissue of origin. Linear regression and mixed-effects models examined associations between tissue origin and physical measures. After neutrophils, vascular endothelial cells (5.3%), erythrocyte progenitors (4.8%), and monocytes (4.3%) were the most frequent cell types of origin. Hierarchical clustering demonstrated three sub-populations distinguished by cf-gDNA tissue origin: Cardiovascular, Erythrocyte Progenitor, and Immune Cell. Individuals in the Cardiovascular sub-group had enrichment of fragments from myocardial and vascular endothelial cells, demonstrated higher frailty scores, and had increased prevalence of myocardial infarction, congestive heart failure, and stroke compared to other sub-groups. The Cardiovascular sub-group also had DNA fragments with increased biological age compared to the other groups (via GrimAge calculation), suggesting the cf-gDNA was released from older cells in the Cardiovascular group. Our study reveals a sub-group enriched with fragments originating from cardiovascular tissue that demonstrate higher future occurrence of frailty and cardiovascular disease and have cf-gDNA release from older cells compared to the other sub-groups. Using cf-gDNA to identify these at-risk individuals can allow for early initiation of treatments to attenuate progression to severe cardiovascular disease and frailty.

